# Towards a definition of an existential approach in cancer patients: a co-produced scoping review

**DOI:** 10.1371/journal.pone.0330384

**Published:** 2025-09-09

**Authors:** Anouk M. de Sain, Carla Ruis, Margot L. Zomers, Marjon E. G. ten Hoor-Suykerbuyk, Theo Kuunders, Filip Y. F. L. de Vos, Astrid Janssens, Tatjana Seute

**Affiliations:** 1 Department of Neurology and Neurosurgery, Brain Center Rudolf Magnus, University Medical Center Utrecht, Utrecht, The Netherlands; 2 Department of Experimental Psychology, Helmholtz Institute, Utrecht University, Utrecht, The Netherlands; 3 Bioethics and Health Humanities, Julius Center for Health Sciences and Primary Care, University Medical Center Utrecht, Utrecht, The Netherlands; 4 Utrecht, The Netherlands; 5 Municipal Health Service Hart Voor Brabant, ‘S-Hertogenbosch, The Netherlands; 6 Department Tranzo, Tilburg School of Social and Behavioral Sciences, Tilburg University, Tilburg, The Netherlands; 7 Department of Medical Oncology, University Medical Center Utrecht, Utrecht, The Netherlands; 8 Department of Public Health, User Perspective and Community-Based Interventions, University of Southern Denmark, Odense, Denmark; Swiss Paraplegic Research, SWITZERLAND

## Abstract

**Background:**

Attention to existential needs has become part of daily treatment. Studies have described the concepts of existential experiences and existential interventions. However, a consensus or conceptual clarity regarding an existential approach in cancer patients is currently missing. This scoping review investigates the term existential approach and suggests a definition.

**Methods:**

We searched PubMed, Embase, Web of Science, and PsycINFO databases until March 2024. We used the Joanna Briggs Institute guidance, and the Preferred Reporting Items for Systematic reviews and Meta-Analyses extension for Scoping Reviews to conduct the review in a systematic manner. The review was conducted in collaboration with patient colleagues who were diagnosed with cancer. Two categories were included: 1) articles investigating elements that are present in existential approaches according to patients and 2) articles investigating elements that are not yet present in existential approaches but recommended by patients.

**Results:**

2791 articles were identified for title and abstract screening, of which 90 were screened for full text. Seventeen articles were eligible for inclusion. Elements were identified that led to the following definition of an existential approach: *‘Medical practice based on a holistic and interdisciplinary view wherein the personal alliance provides room for mutual sharing of thoughts, acknowledging existential issues besides medical issues, whilst paying attention to personal values and what matters most’.*

**Conclusions:**

This definition provides a framework and therefore makes an existential approach more applicable. Different healthcare providers (e.g., nurses/physicians) can be part of an existential approach. To provide an existential approach in daily treatment, future studies should focus on training in communication skills.

## 1. Introduction

Cancer and, unintentionally, cancer treatments can result in a shortened life span, persistent fatigue, and changes in appearances, such as hair loss [1–3].

Moreover, cancer can affect someone’s perspective of the world and their sense of physical, psychological, social, and spiritual ‘being’ [4], as receiving the diagnosis of cancer can evoke existential concerns in patients [4–7]. Previous literature defines existential needs as ‘the necessity of experiencing life as meaningful’ [8]. It is important to recognize the potential existential needs of cancer patients as neglected existential distress has been linked to a reduction of quality of life and an increase of anxiety and depression [9].Acknowledging the potential existential needs of cancer patients is also of importance in the context of the developing patient-doctor relationship. In the past, the relationship between doctor and patient could be described as a patient who is seeking for help and a doctor whose decisions were unquestionably followed by the patient [10]. To date, there is more attention for a patient-centred approach and shared decision making with the patient being an active partner in their healthcare [11]. An increasing amount of literature suggests that doctors are able to play a role in addressing the existential needs of patients [12,13], yet in practice many doctors seem to focus on medical rather than on personal, existential aspects in their conversations with patients [7,14,15]. Parallel to this shift towards person-centred care in health care, a similar change can be noted in the health research where patients are being engaged in the research process. Involving patients in health research, advocated by the revised Helsinki Declaration [16] can lead to more relevant (patient-centred) research, research of greater quality, and make it more effective, efficient, and more sustainable [17–19]. Research in collaboration with patients produces research findings that address patient and public concerns [20].

The neuro-oncology department of the University Medical Center Utrecht works from an interdisciplinary and holistic perspective with attention to the existential well-being of patients. Healthcare professionals from this department learn from their patients that a holistic approach leads to a well described context in which the patient functions as a multidimensional being. Moreover, they learn the importance of taking the existential well-being of patients into account. Attention for existential needs has therefore become part of the day-to-day treatment and decision making at this department. That is to say, it is incorporated into daily practice and not a separate intervention or therapy for which one needs an appointment with specifically trained personnel (e.g., a psychologist, psychiatrist or spiritual caregiver). We are studying this so-called “existential approach” and as part of this study we aim to review what is written in literature on an existential approach in cancer patients.

While there are reviews on existential concerns in cancer patients, and on interventions or therapies targeting existential concerns in cancer patients, there is to date no review published on the incorporation of attention for existential needs in daily practice, i.e., an existential approach from a broader perspective. A previous review of Henoch and Danielson [21] provides an overview of the meaning of existential concerns in cancer patients and related interventions, such as psychotherapy and cognitive existential group therapy. Furthermore, the review of Tarbi and Meghani [22] describes the concept of existential experience of adults with advanced cancer as “a dynamic and fluid state, preceded by confronting mortality and becoming aware of death”. However, the review of Tarbi and Meghani [22] focuses on the existential experience and not the definition of an existential approach in cancer patients. In addition, the non-systematic review of Wexler and Corn [23] describes an existential approach in cancer patients. This approach suggests an expansion of the role of the oncologist, with a greater emphasis on holistic aspects of cancer care. The systematic review of Bauereiß and colleagues [24] looked at existential interventions in patients with cancer, where they stated that an existential intervention had to be ‘manualized and address existential needs as a main component’. However, they only included randomized controlled trials. Moreover, an extensive amount of their included studies include interventions and specific therapies (e.g., psychotherapy) performed by skilled/trained personnel (e.g., a psychologist). Our review therefore suggests a broader theoretical framework. We propose the term ‘approach’. Instead of a separate intervention or therapy for which one needs an appointment with skilled/trained personnel (e.g., a psychologist, psychiatrist or spiritual caregiver), we suggest that an approach includes what it entails to incorporate attention for existential needs into the day-to-day treatment and decision making.

The main objective of our review was to search for descriptions of ‘an existential approach in cancer patients’ and to formulate a comprehensive definition. The following research question led the review: *How can an existential approach in cancer patients be described*? Given the fact that we formulated a relatively broad research question, we conducted a scoping review. A scoping review is useful in exploring emerging evidence with a broader “scope” and to clarify definitions that are used in literature [25]. This scoping review was co-produced with patients with cancer, which means that they were actively involved in the review and writing process. The involvement of patients in a review process has, among others, shown to be beneficial in designing strategies and identifying relevant information [26].

## 2. Methods

For this review, we used the framework of Arksey and O’Malley [27] which consists of five stages: 1) identifying the research question, 2) identifying relevant studies, 3) study selection 4) charting the data, 5) collating, summarizing and reporting the results. We used the latest Joanna Briggs Institute (JBI) guidance for scoping reviews [28] and the PRISMA-ScR (Preferred Reporting Items for Systematic reviews and Meta-Analyses extension for Scoping Reviews) checklist to describe our results [29] in a systematic manner. The PRISMA-ScR checklist can be found in S1 appendix. We chose the Arksey and O’Malley’s framework because of its fundamental role in structuring scoping reviews. To strengthen the methodological transparency, we combined the framework with recent JBI guidelines.

### 2.1 Patient involvement

This scoping review was conducted in collaboration with two patients from the neuro-oncology department from hospital the neuro-oncology department from the UMC Utrecht (UMCU). Both patients (TK and MHS), who are currently under treatment at the hospital, are involved as patient colleagues in the PhD-project of the first author and help in the design of the study and writing process. Both patients have an academic background and a PhD. At the start of this study, we discussed the planned work and their possible contributions and it was decided that they would join the review process from full text screening onwards, given the time investment required to be engaged from the start.

### 2.2 Data sources and searches

Prior to the formulation of the search string, we have attempted various search strategies, using terms such as existential approach, existential framework and existential treatment. These searches failed to capture important studies known to the research team. Therefore, we kept the search strings broad and opted to screen a larger amount of records on title and abstract. The search strategy was developed in consultation with a librarian from Utrecht University (UU). A search was performed in PubMed and CINAHL to identify key articles on the topic. The keywords and related Medical Subject Headings (MeSH) from the titles and abstracts of these articles were identified to develop a search strategy for the review. We conducted our search in de following databases: PubMed, Embase, Web of Science and PsycINFO. The databases were selected based on comprehensiveness and relevance. The search strings we used can be found in S2 Appendix. The search was performed on the 5^th^ of March 2024. In case an article was not open access, we requested the article from the UU library. If the article was not available, an email was sent to the corresponding author. If no response was received from the author, this article was labelled as not available.

### 2.3 Inclusion and exclusion criteria

An overview of the inclusion and exclusion criteria can be found in Table 1. This review included English peer-reviewed original articles. Letters to the editors, reviews, conference abstracts, book chapters, protocols and editorials were excluded. There was no limitation in publication time of the articles. The inclusion criteria were adult patients (18+) with a cancer diagnosis (all types and stages, both palliative and non-palliative patients). The aim was to define an existential approach in cancer patients thus studies with cancer survivors (free of cancer) were excluded. Only information from the patient perspective was extracted from articles with study populations including patients, caregivers and healthcare providers, since patients experience potential existential needs in in the disease trajectory. We excluded articles that solely focused on the perspective of healthcare providers or/caregivers. All settings (e.g., hospital, nursing home) were included. The articles were included if they focused on an existential approach/framework for daily practice/ treatment and excluded when they focused on a specific intervention or therapy (e.g., psychotherapy), performed by skilled/trained personnel. Studies solely focusing on spiritual/religious care/end-of life care without existential components were not considered as part of an existential approach and therefore excluded. Two categories of articles were included: 1) articles describing elements that are already present in existential approaches according to patients and 2) articles describing elements that are not yet present in existential approaches but recommended by patients.

**Table 1:** Inclusion and exclusion criteria.

Study characteristics	Inclusion criteria	Exclusion criteria
*Publication type*	English peer-reviewed original articles.	Letters to the editors, reviews, conference abstracts, book chapters, protocols and editorials.
*Publication time*	No limitation in publication time of the articles.	
*Population and perspectives*	Adult patients (18+) with a cancer diagnosis (all types and stages, both palliative and non-palliative patients). Information from the patient perspective was extracted from articles with study populations including patients, caregivers and healthcare providers.	Cancer survivors (free of cancer). Articles that solely focused on the perspective of healthcare providers or caregivers.
*Outcome*	Focus on an existential approach/ framework for daily practice/ treatment.	Focus on a specific intervention or therapy (e.g., psychotherapy), performed by skilled/trained personnel. Studies solely focusing on spiritual/religious care/end-of life care without existential components.
*Settings*	All settings (e.g., hospital, nursing home).	

### 2.4 Evidence screening and selection

All literature references retrieved through the initial search were uploaded to Zotero version 6. The articles were then saved in the web-tool Rayyan and deduplicated. Rayyan was chosen since it was user-friendly for the (patient)-colleagues during the screening process. During the title and abstract screening, the first 150 articles were screened by AJ, AS and CR. Discrepancies and uncertainties were discussed. AS screened 100% of the articles on title and abstract. During this title and abstract screening process, we mainly focused on the exclusion criteria. During the full text screening, more than 60% of the articles were divided and double screened by CR, AJ, and patient-colleagues TK and MHS. AS screened again 100% of the articles. Interrater agreement was above 90%. In case of disagreement on inclusion, TS was consulted. AS extracted the data using a bespoke template including author, title/year, target population, measurements and elements of an existential approach.

## 3. Results

### 3.1 Study characteristics

**Fig 1:**
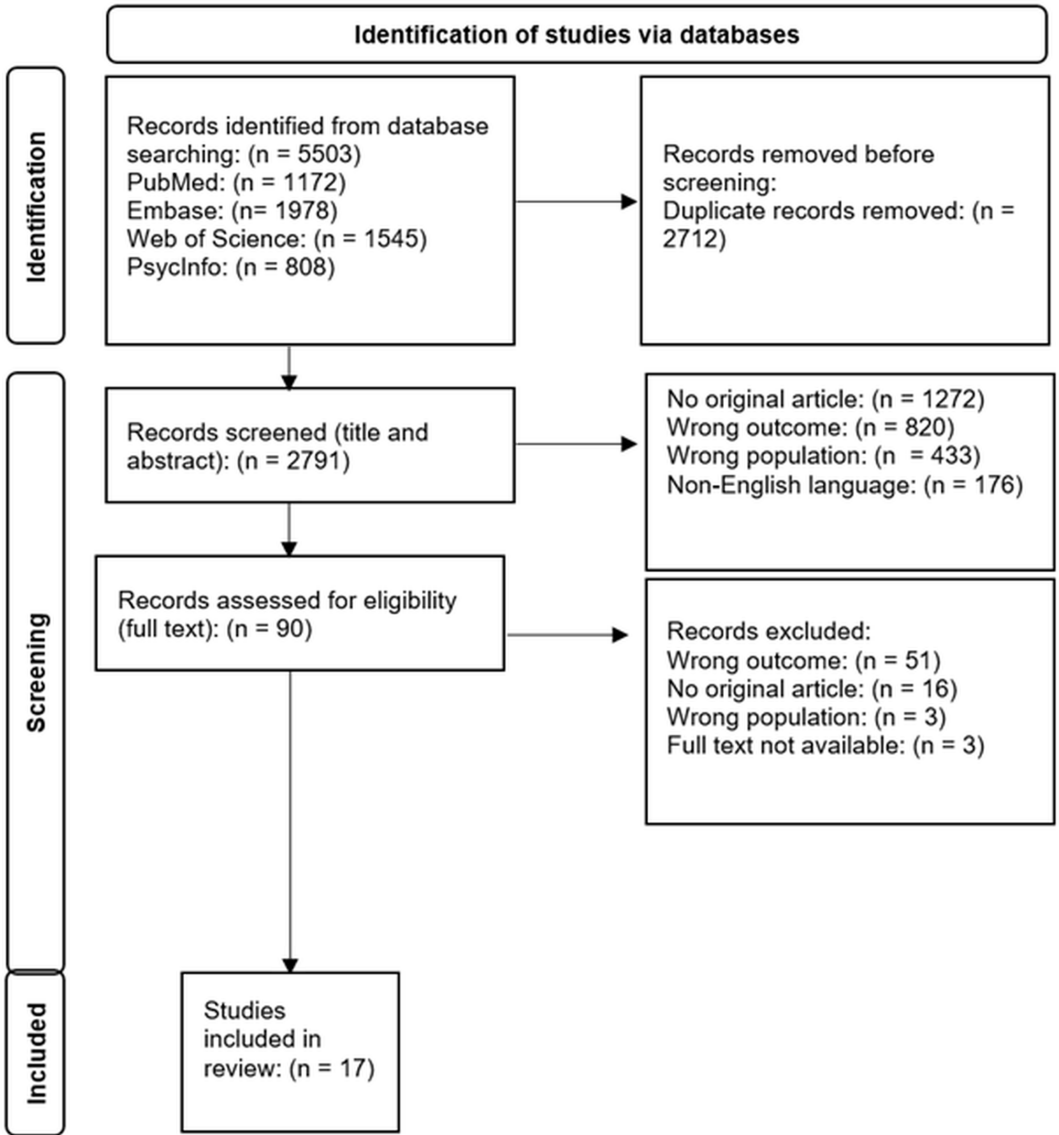
Inclusion flow diagram.

The initial search yielded 5503 articles. 2791 articles remained for title and abstract screening after the removal of duplicates. Ninety articles were screened on full text and 17 studies were included in the review. Fig 1 shows the PRISMA flow diagram of the search process. The most common reason for exclusion during the title/abstract phase was that the articles did not report an original study. This was followed by: *outcomes not related to an existential approach*, *wrong population*, *non-English language* and *article not available.*

### 3.2 Study and population characteristics

*Studies describing elements that are already present in existential approaches according to patients*

Twelve studies described elements that are already present in existential approaches according to patients. Seventy-five percent of the included studies were conducted in Europe: Denmark (n = 4), Sweden (n = 1), United Kingdom (n = 1), Norway (n = 1), Germany (n = 1) and Switzerland (n = 1), and 25% in the USA (n = 3). The 12 studies included 11 different samples. The studies of Myrhøj [30,31] involved the same patient group. The year of publication ranged from 2001–2024. A qualitative research design was mostly used (n = 10). One quantitative study and one case study were included. A wide range of cancer types were represented in the studies, including (non-small cell) lung, gastrointestinal, colorectal, colon, breast, prostate, brain (glioma), multiple myeloma, hairy cell leukaemia, lymph/blood, urinary tract, skin or non-specified (advanced cancer, stage III/IV). In the majority of the articles, only patients were subject of the study (n = 8), other studies involved patients and caregivers and/or healthcare providers (n = 4).

*Studies describing elements that are not yet present in existential approaches but recommended by patients*

Five studies described elements that are not yet present in existential approaches but recommended by patients. All of which were performed in Europe: Norway (n = 2), Sweden (n = 2), and Switzerland (n = 1). The year of publication ranged from 2006–2022. All the studies have a qualitative design. Patients had various forms of (advanced) cancer, including head and neck, prostate, breast gastric, colon, lung, multiple myeloma, pancreatic, kidney cancer. Three studies included conversations with healthcare providers and patients, two studies focused solely patients.

Table 2 presents the elements of an existential approach as identified from the included studies.

**Table 2:** Elements existential approach.

*Elements that are present in existential approaches according to patients*			
Article title	Target population	Measurements	Elements existential approach (cited from articles)
“I Had a Lot More Planned”: The Existential Dimensions of Prognosis Communication with Adults with Advanced Cancer [32]	A subset of the Palliative Care Communication Research Initiative (PCCRI) parent study. 34 patients with a primary life-limiting illness of metastatic cancer (lung, gastrointestinal (non-CRC), CRC/breast/prostate and other)	Audio-recorded and transcribed inpatient palliative care conversations [45] between adults with advanced cancer, their families, and palliative care clinicians	*Conversations with more discussion of prognosis also contained more discussion of existential topics. This represented an existential shift in focus, from an emphasis on symptom management, and pain management specifically, in conversations with no prognosis communication to an emphasis on time and “what matters most” in conversations with more prognosis communication.*
The existential cancer journey: Travelling through the intersubjective structure of homeworld/alienworld [33]	Cancer patients. 11 participants for semi-structured interviews and 46 participants for 9 focus groups	Nine weeks of participant observation and informal interviewing at a Danish rehabilitation centre, 11 interviews and 9 focus group interviews	*A way to respond to apparent existential distress and needs of cancer patients and survivors is to address and communicate with patients about possible existential questions, reflections and concerns related to feelings of meaninglessness, hopelessness, disruption, anger, loneliness, fear and suffering and death as well as communicating with patients about whether and how they feel existentially ‘at home’ in their present lifeworld.*
Existential support in brain tumour patients and their spouses [34]	20 brain tumour patients, 16 family members and 16 nurses	Explorative, tape-recorded, semi-structured interviews about existential issues	*Conversation- about the existential threat, comfort, Holistic way of thinking, and being seen.*
Interdisciplinary collaboration in serious illness conversations in patients with multiple myeloma and caregivers – a qualitative study [30]	22 patients (with multiple Myeloma) and caregivers, four nurses, four physicians	11 dyadic interviews, two focus groups	*Interdisciplinary collaboration created a more holistic conversation, as it combined medical issues with existential considerations related to the patients’ everyday life and what mattered most to them and their caregiver.* *Interprofessional collaboration during serious illness conversations can promote a holistic and existential approach, while also strengthening the integration of palliative care within the context of haematological disease trajectories. The use of both existential and descriptive language within the same conversation reflects the interdisciplinary interaction, which strengthens each professionals’ expertise and improves the quality of patient care.*
Serious illness conversations in patients with multiple myeloma and their family caregivers—A qualitative interview study [31]	12 patients with multiple myeloma and 11 caregivers	Interpretive phenomenological analysis	*Increasing dyadic communication- when healthcare providers have an existential rather than a medical language, patients will strengthen and expand their existential linguistics.* *Having adequate amount of time contributed to discussions of more existential values.*
The Challenge of Existential Issues in Acute Care: Nursing Considerations for the Patient With a New Diagnosis of Lung Cancer [35]	73 men and women with a diagnosis of non-small cell lung cancer	Semi-structured interviews	*Identifying strategies that foster resolution of existential and death-related concerns in the early postdiagnostic period and during the acute treatment process is essential. Nurses who are comfortable with listening for and discussing existentially related concerns during the acute phases of treatment may be in a better position to promote the patient’s psychological adaptation.*
Social, psychological and existential well-being in patients with glioma and their caregivers: a qualitative study [36]	26 patients with suspected glioma, 21 received a confirmed diagnosis of glioma and only these patients participated in further research. 23 of the patient relatives 20 general practitioners	Interviews	Practical suggestions regarding social, psychological and existential well-being: *At referral: acknowledge the distress of uncertainty while waiting for a diagnosis* *Around diagnosis: offer more detailed information about the likely process of what will happen and the expected diagnosis* *During initial treatment: provide an opportunity for patients to voice their problems or questions, give supportive advice to caregivers and allow them to ask questions, ensure immediate communication between the hospital and primary care facility* *At follow-up: identify a named person (possibly a specialist nurse or family physician) who will maintain contact and offer psychological support and practical advice,* *discuss what the future may hold and possible symptoms, involve caregivers in discussions about practical help, where appropriate* *Disease progression: sensitively give patients the opportunity to plan their future care, and to consider options such as their resuscitation preferences, living wills and their preferred place of death, involve caregivers in discussions about end-of-life care (with patients’ consent), communicate the patient’s care plan with all other services, including after-hours services, maintain contact and support the caregiver* *Bereavement: provide an initial bereavement visit from a known and key professional* *and offer further contact.*
Older Adults’ Experience of Meaning at the End of Life in Two Danish Hospices: A Qualitative Interview Study [33]	10 patients aged with a primary diagnosis of cancer	Semi-structured interviews	*Entering into an existential dialog with people, and by being aware of not primarily using the medical vernacular when talking with primary ill people. This study suggest that healthcare professionals also should apply existential and not just medical vernacular when addressing terminally ill people, because this may strengthen their experience of meaning and hope in the face of death.*
A 60-year-Old Male with Hairy-Cell Leukemia and Existential Distress [34]	One 60-year-old man with hairy-cell leukaemia	Case study	*Fully recognizing and maximizing the power and value inherent in a meaningful, collaborative therapeutic alliance between the patient and the therapist, the alliance itself becomes an effective and efficient clinical instrument to address certain constructs of existential distress such as a meaninglessness and isolation.*
Perceived doctor‐patient relationship and its association with demoralization in patients with advanced cancer [37]	187 patients with stage III or IV cancer	The German quality of Life at the End of Life‐Cancer‐Psychosocial questionnaire (QUAL‐EC‐P), which is derived from the Quality of Life at the End of Life‐Cancer Questionnaire (QUAL‐EC)	*The importance of the physician‐patient relationship in the context of advanced cancer. The perception of how doctors behave and communicate has great relevance for the patient’s ability to cope with existential distress at the end of life.*
Compassionate Presence in Seriously Ill Cancer Patients [38]	50 patients with advanced cancer	A subscale of the QUAL‐EC‐P for assessing doctor‐patient relationship	*Compassionate presence (existential togetherness). Compassionate presence often induced mental-spiritual changes in patients who were cared for by professionals. Patients described compassionate presence as a relational quality.*
The illness reframing process in an ethnic-majority population of older people with incurable cancer: variations of cultural- and existential meaning-making adjustments [39]	21 patients with cancer (colon, prostate, female breast, lung, lymph/blood, urinary tract, skin)	Semi-structured interviews	*Having a multi-dimensional framework, and seeing the framework content in a process-oriented perspective.* *The importance of cultural analysis for patient care, and for understanding the existential dimension in the cultural framework as pivotal for negotiating changes made in other dimensions.* *Information gained through cultural- and existential meaning-making analysis at the individual level, supported by cultural information at the national and local context levels.*
***Elements that are not yet present in existential approaches, but recommended by patients***			
Physicians’ responses to advanced cancer patients’ existential concerns: A video-based analysis [7]	13 patients and 5 physicians. The patients had advanced cancer	Video-recording	*Physicians should be attentive to their possible habit of steering the agenda towards biomedical topics, hence, avoiding patients’ existential concerns. Initiatives like tools and course programs cultivating behaviour that are known to enhance person-centred and existential communication should be implemented in clinical practice and medical training to promote coping, autonomy, and existential health. Video recordings of conversations could be used in quality improvement for example in reflection groups for health care personnel. When appropriate, the physician could invite others within the interdisciplinary team to provide expertise in existential and emotional support.*
What symptoms tell us: A multiple case study of oncology consultations [40]	24 oncology physicians and 124 patients with advanced cancer. The study mentions 5 cases	Audiotaped consultations	*A supportive response, consisting of a statement that the physician is affected by the patient’s suffering, recognizing his own impotence and inability to help, would here also show the family, who has turned their own impotence into an aggressive denial of the patient’s distress.*
“Eh – What type of cells are these – flourishing in the liver?” Cancer patients’ disclosure of existential concerns in routine hospital consultations [4]	Consultations between healthcare providers and patients with advanced cancer (n = 13). Cancer types were: head, neck, prostate, gastric, colon, lung, myelomatosis, pancreatic, kidney and various types of metastases	Video-recordings	*Health professionals should be attentive to underlying existential messages embedded in the patient’s questions and concerns. Acknowledging these existential concerns provides an opportunity to briefly explore the patient’s needs and may direct how the physician tailors information and support to promote coping, autonomy, and existential health.*
Lived experiences and caring needs in young adults diagnosed with cancer [33]	8 females with cancer	Qualitative analysis of narratives	*For this group of young people with cancer, it is essential to offer tailored, personal relationships, effective communication, and trust building through direct contact with a nurse. There is a need in nursing education to address existential matters, to make nursing students ready to be approached by such questions in their professional work. Also, we believe that the experienced nurse must be given means to feel confident to handle questions about existential matters. The nurse has to have the courage to meet and challenge the vulnerability in this group of cancer patients. This can only be possible if the nurse has the opportunity of regular, critical reflection with a supervisor. Collaboration in teams and with support groups will make nursing care more comprehensive in the future. Being presented with the possibility of a regular group session both within the same profession but also with other professions can be positive for the participants in the group sessions.*
Life, illness and death—Existential reflections of a Swedish sample of patients who have undergone curative treatment for breast or prostatic cancer [36]	10 patients (5 with breast cancer, 5 with prostate cancer) who received curative treatment	Interviews	*The basic task in existential support is to meet another person face to face and listen. For this task courage and training in communication are needed. What nurses need is to increase their knowledge and awareness of existential issues as well as make it a priority to use time to meet their patients’ existential issues.*

*Elements that are already present in existential approaches according to patients*

### 3.3 Attention for existential topics in addition to medical topics

Several included studies reported that patients indicated that their healthcare providers paid attention to existential topics in addition to medical topics [30,32,41]. Another study describes the use of an existential language (emphasizing wishes, goals, fears and worries) rather than discussing medical subjects [31]. In addition, existential conversations could include receiving help to cope with the threat of death, talking about and acceptance of death [35].This extensive collaborative conversation allows for a more holistic conversation, in which the overall picture of the person is addressed. This holistic view is also stressed in other studies [30,35].

### 3.4 An interdisciplinary existential dialogue: engaging as a healthcare provider and addressing personal values

One of the studies indicated that patients consider an interdisciplinary dialogue in which with both nurses and physicians are present as element of an existential approach, because in these dialogues a combination of medical conditions and existential considerations is addressed [30]. Emphasizing the existential shift leads to conversations about time (good times/hard times) and “what matters most” for patients, instead of focusing on physical symptoms [30,32]. Moreover, it is suggested to engage in existential dialogues as healthcare provider [41]: a mutual sharing of thoughts, worries, hope and uncertainties between healthcare provider and patients helps in entering a dialogue about patients’ believes about life beyond death.

### 3.5 Compassionate presence and the use of therapeutic alliance

One of the studies discusses compassionate presence (mentioned as “existential togetherness”) as element of an existential approach [38]. The patients describe compassionate presence as a relational quality that stimulated mental-spiritual alterations. Another element of an existential approach is the alliance between patient and therapist [34,37]. The collaboration within the relationship of a patient and healthcare professional can be used as a tool to address existential distress [34]. Furthermore, the patients emphasize *comfort* and *being seen* as part of what can be defined as existential support. They describe the importance of *presence* of the healthcare providers and physical touch [35]. According to qualitative interviews with patients, healthcare providers respond to existential distress by addressing “*possible existential questions, reflections and concerns related to feelings of meaninglessness, hopelessness, disruption, anger, loneliness, fear of suffering and death as well as communicating with patients about whether and how they feel existentially ‘at home’ in their present lifeworld*” [42]

### 3.6 Cultural-clinical aspects

An existential dimension of a patient can be better understood, if a cultural analysis is taken into account [39]. By taking existential and personal values into account, we can better understand how patients understand and cope with their disease.

### 3.7 Other important elements described by patients: practical suggestions during the disease journey

Cavers and colleagues [43] highlighted practical suggestions in their existential, social and psychological approach. Their suggestions acknowledge uncertainty before receiving a diagnosis, providing detailed information, and facilitating communication among caregivers. Furthermore, it is important to have a contact person and to communicate about end-of-life care. Lastly, it is essential to focus on existential aspects early on: in the postdiagnostic phase and during the treatment period [44].

*Elements that are not yet present in existential approaches but recommended by patients*

### 3.8 Attention for existential topics and medical topics

Physicians should be aware of their possible habit leading the conversation towards biomedical topics and avoiding existential concerns [4]. Both biomedical topics and existential concerns should be discussed [7]. Physicians should acknowledge these existential concerns and suffering [4] show that they are supportive [40].

### 3.9 Communication training and support by interdisciplinary team

Training in communication skills [7,36] or support (existential as well as emotional) by an interdisciplinary team [7] should be provided.

### 3.10 A personal relationship

For the patients, access to a personal relationship with a member of the health care team, preferably with a trained nurse is recommended [36,33]. Listening and sufficient knowledge about existential issues are also mentioned [36].

### 3.11 Definition existential approach

Based on the synthesis of the elements described in the studies we propose the following definition of an existential approach:

**‘Medical practice based on a holistic and interdisciplinary view wherein the personal alliance provides room for mutual sharing of thoughts, acknowledging existential issues besides medical issues, whilst paying attention to personal values and what matters most’**

## 4. Discussion

We conducted a scoping review searching for studies that described an existential approach in cancer patients. These studies offered an insight in what can be considered as an existential approach and a broad overview of elements of an existential approach in cancer patients. Based on the synthesis of these elements we developed a definition of an existential approach in cancer patients.

Elements in an existential approach were attention for both existential and medical topics [30–32]. One of the studies indicated that patients consider an interdisciplinary dialogue with both nurses and physicians present as important for an existential approach, because these dialogues allow for a combination of medical conditions and existential considerations to be addressed [30]. It is suggested to engage in a two-way existential dialogue (healthcare provider and patient) through mutual sharing of thoughts, worries, hopes and uncertainties [41]. Multiple studies describe a holistic view as the basis for an existential approach [30,41,35]. Emphasizing the existential shift leads to conversations about time (good times/hard times) and “what matters most” for patients, instead of focusing on physical symptoms [30,32] Moreover, presence, being seen as a person, and the alliance between patient and healthcare providers are considered important [35,38,37] It is also important to focus on existential aspects early after initial diagnosis and during the first treatment period [44].The studies discussed above show an overlap with another group of studies that offered elements regarding an existential approach. However these patients expressed what they are currently missing and what they would like to see integrated in an existential approach. Elements were attention for existential topics and medical topics [4,7,40]. Moreover, communication training and support by an interdisciplinary team is recommended [7,36]. Lastly, a personal relationship, listening and sufficient knowledge about existential issues are addressed [36].

### 4.1 Challenges in practicing an existential approach

Although the importance of a shift from discussing medical topics to an existential dialogue is frequently mentioned, articles included some critical notes [30,41] In particular, it is described that the language of healthcare providers can be a challenge to practice an existential approach, as their language is often rooted in solution-oriented medical jargon [41]. Viftrup and colleagues [41] therefore emphasized the need for communication techniques and how communication influences the quality of life of patients nearing the end of life. Moreover, Myrhøj and colleagues [30] showed that it is primarily the nurse who addresses existential aspects and the physician who focusses on medical issues. For this reason, Myrhøj and colleagues [30] suggested interdisciplinary consultations, integrating the attendance of both a nurse and physician. In this way, extensive conversations on both existential- and medical issues can be discussed. This aligns with the study of Hvidt [42] which suggested that future research should focus on improving communication skills in healthcare providers. In contrast to the suggestion of an interdisciplinary framework, another study only addressed the role of the nurse and not from other perspective (e.g., doctor) [44].

Other obstacles regarding practicing an existential approach were mentioned. Patients in the study of Strang and colleagues [35] described that they felt that their healthcare providers experience a lot of stress and are therefore not approachable for support regarding existential questions. Patients felt as if the healthcare providers did not have sufficient time and therefore did not want to bring in existential questions. Moreover, a part of the patients indicated that the healthcare providers did not have the knowledge to provide existential support [35]

### 4.2 Multidisciplinary approach

It is important to involve multiple healthcare providers (e.g., doctor/nurse) during the disease trajectory to ensure broad applicability and to make sure existential themes are being discussed. Previous research of Wexler and Corn [23] describes an existential approach in cancer patients in a non-systematic review. In their discussion, they advise an expanded role for the oncologist, which can be described as a more holistic approach. They recommend a training to obtain the required skills, such as communicating skills. The results of their review are partially consistent with the findings of our scoping review, since we also suggest a holistic perspective. However, the added value of our study, compared to Wexler and Corn [23]is that we clarify what an existential approach entails and provide a clear definition of team-orientated existential approach.

### 4.3 Clinical implications: Synthesis of the results

The synthesis of the results from this scoping review led to the following definition: ***‘Medical practice based on a holistic and interdisciplinary view wherein the personal alliance provides room for mutual sharing of thoughts, acknowledging existential issues besides medical issues, whilst paying attention to personal values and what matters most’.*** In line with the previously mentioned shift towards person-centred care, attention for and addressing existential needs has become part of day-to-day treatment in the department neuro-oncology at the UMCU. Therefore, we suggest that this definition should be extended to and include its application, and thus suggest the term *existential treatment*.

### 4.4 Strengths and limitations of the study

This scoping review has addressed an important research question using an extensive systematic search of the literature. The included databases cover a relevant portion of the literature and therefore it is unlikely that further databases would have provided additional results [45,46]. To our knowledge, this is the first systematic search for a description of an existential approach in cancer patients. Another strength is the involvement of patient colleagues during the full-text screening and writing process, which has contributed to identifying relevant information regarding the definition of an existential approach and their contribution has helped in the writing process. The patient colleagues helped formulate the definition and checked whether it matched their actual experiences. Our scoping review also has some limitations. Firstly, we decided to only include papers published in English. Secondly, the included studies of this review were from Europe, which involved countries with a predominantly white population, and the USA. Therefore, one might question whether an existential approach is a Western term and whether or not is included/ named differently in non-Western countries. Another limitation of the study is the exclusion of studies focusing solely on healthcare professionals. For example, the nurses in the study of Strang and colleagues [35] described an existential approach as: *religion, diffuse picture, conversation and questions of vital importance.* The patients of the study [35], by contrast, did not mention religion. A suggestion as to why patients still report unmet existential needs, could be because the existential needs are viewed differently by healthcare professionals. Moreover, it is also reasonable that there are different ways to describe an existential approach, since patients have different existential needs [47].

### 4.5 Implications for clinical care

This review aimed to describe an existential approach in cancer patients. The proposed definition provides a framework and therefore makes an existential approach more applicable. Different healthcare providers (e.g., nurses/physicians) can be part of an existential approach. However, patients do not always feel as if the healthcare providers have sufficient knowledge to discuss existential topics. To provide an existential approach, various studies suggest training, for example in communication skills [7,36]. A tool to learn these communication skills could be helpful to train healthcare providers in delivering existential treatment.

### 4.6 Implications for future research

This study proposed a definition regarding an existential approach in cancer patients. Only information from the patient perspective was extracted from articles, since patients have existential needs in their disease trajectory. Building on these findings, future work could explore whether a definition of an existential approach differs or aligns for patients, significant others and healthcare providers. Our results showed that elements of an existential approach are not always present but are recommended by patients. Therefore, future research should examine the applicability and integration of the definition within healthcare.

## Supporting information

S1 Appendix PRISMA-ScR checklist. (PDF)

S2 Appendix Search strings and strategy. (PDF)

S3 Table Title/abstract files. (XLSX)
