# Supplementary material for: Towards a definition of an existential approach in cancer patients: a co-produced scoping review
Source: PLoS One. 2025 Sep 9;20(9):e0330384. doi: 10.1371/journal.pone.0330384 (PMC12419588; doi:10.1371/journal.pone.0330384)
Supplement: S2 Appendix — (PDF) [file pone.0330384.s002.pdf]

S2 Appendix: search strings and strategy

Pubmed

(“existential”[Title/Abstract]) AND (“Neoplasms”[Mesh] OR “neoplasm*”[Title/Abstract] OR “oncology”[Title/Abstract] OR “cancer*”[Title/Abstract] OR “tumor*”[title/abstract] OR “tumour*”[Title/Abstract])

EMBASE

('existential':ti,ab,kw) AND ('neoplasm'/exp OR 'neoplasm*':ti,ab,kw OR 'oncology':ti,ab,kw OR 'cancer*':ti,ab,kw OR 'tumor*':ti,ab,kw OR 'tumour*':ti,ab,kw)

Web of Science

(TS=(existential)) AND TS=(neoplasm* OR oncology OR cancer* OR tumor* OR tumour*)

Psychinfo (OVID system)

APA PsycInfo <1806 to February Week 5 2024>

1	exp neoplasms/63447	
2	existential.ab,id,ti.	13812
3	(neoplasm* or oncology or cancer* or tumor* or tumour*).ab,id,ti.	92895
4	1 or 3	96500
5	2 and 4 808	

<u>Strategy during title/abstract screening</u>

1. Is the article an original article? (for example: no conference abstract)
2. Is the article in English?
3. Is the population correct? (patients with cancer and 18 years and older)
4. Is it possibly relevant or not (for example: is it a specific therapy? → exclusion criteria)?

During screening, we focus particularly on the exclusion criteria. If it was uncertain whether the population was incorrect (e.g., patients with a life limiting disease), but potentially interesting, the was included and the population was checked during the full text screening.

<u>Strategy during full text screening</u>

1. Is the population correct? → cancer patients. This scoping review will look from the patient perspective.
    a. We include the articles with multiple samples (e.g. 50% cancer patients, 50% heart disease) and look at the statements that are made about the cancer patients.

Important aspects:

What we are not looking for

- We are not looking for articles that examine existential suffering/existential concerns in the cancer population and only describe at the end of the article: ... so there should be attention to this existential suffering or caregivers should pay more attention to this. This is not related to the research question.
- We are not looking for a definition of spiritual care.
- We are not looking for the meaning of end-of-life care.

What we are looking for

- We are looking for an existential approach and not a specific therapy. By this we mean that the approach should be able to be implemented by any healthcare professional (so not, for example, only by psychologists).
- We are looking for an existential approach. With this we mean, are certain skills required for the healthcare professionals? How do we provide existential care? What are important aspects? What should the emphasis be?
